# Cloning and expression analysis of *VrNAC13* gene in mung bean

**DOI:** 10.1515/biol-2022-0627

**Published:** 2023-07-06

**Authors:** Siyu Zhang, Jing Ai, Yaning Guo, Yu Bai, Han Yao, Fugang Wang

**Affiliations:** School of Life Sciences, Yulin University, Yulin, P. R. China

**Keywords:** mung bean, *VrNAC13*, gene cloning, bioinformatics analysis, qRT-PCR

## Abstract

To explore the role of NAC transcription factors in mung bean (*Vigna ratiata*), we here comprehensively analyzed *VrNAC13* structure and expression patterns in the mung bean cultivar “Yulin No.1”. The nucleotide sequence of *VrNAC13* (GenBank accession number xp014518431.1) was determined by cloning and sequencing the gene. A predicted transcriptional activation domain in *VrNAC13* was validated with a yeast one-hybrid assay. The composition and functional characteristics of *VrNAC13* were analyzed using basic bioinformatics techniques, and the expression characteristics of *VrNAC13* were analyzed via quantitative reverse transcription-PCR. The results showed that *VrNAC13* was 1,068 bp in length and encoded a product of 355 amino acids. *VrNAC13* was predicted to contain a NAM domain and to belong to the NAC transcription factor family. The protein was hydrophilic and contained several threonine phosphorylation sites. Phylogenetic analysis showed that *VrNAC13* was highly similar in sequence to two *Arabidopsis thaliana* NAC proteins; we hypothesize that *VrNAC13* may perform functions in mung bean similar to those of the two closely related proteins in *Arabidopsis*. Promoter analysis of *VrNAC13* revealed *cis*-acting elements predicted to respond to abscisic acid (ABA), gibberellin, auxin, light, drought, low temperature, and other stressors. *VrNAC13* was most highly expressed in the leaves and expressed at very low levels in the stem and root. It was experimentally determined to be induced by drought and ABA. Based on these results, *VrNAC13* appears to regulate stress resistance in mung bean.

## 1 Introduction

The NAC family of transcription factors is a very large family that is found only in plants [1,2]. The family name is derived from those of several family members, namely NAM, ATAF1/2, and CUC1/2. Structurally, NAC transcription factors consist of a conserved N-terminal protein domain and a variable C-terminal transcriptional activation domain. The N-terminal domain contains five subdomains: A, B, C, D, and E. The structures of these subdomains are associated with nuclear localization and with the recognition and binding of downstream target gene sequences. The C-terminal domain is subject to transcriptional activation or inhibition [3]. Numerous studies have found that NAC transcription factors play important roles in plant growth, development, and stress responses and resistance [4,5,6].

Mung bean (*Vigna ratiata* L.) is an important crop plant in China. It is a popular dual-purpose crop for both food and medicine because it is rich in protein, vitamins, mineral elements, and other nutrients in addition to having medicinal value [7,8]. Mung bean is not only economically valuable and rich in nutrition, but it is also relatively hardy; it is drought resistant, requires minimal nutrient input, fixes nitrogen, and is suitable for intercropping with other plant species [9]. The Yulin mung bean production area has a unique environment with clean soil and air, sufficient light and heat resources, and high temperature variation between day and night; mung bean seeds from this area are large and have good color, strong germination potential, and are thus referred to as “green pearls” [10,11]. The Yulin area is located at the south edge of the Maowusu sandy land, which has less annual rainfall (mainly in July and August) and little rainfall from May to June. As a result, drought during the seedling stage is a key factor limiting the development of the mung bean industry in Yulin [12,13]. It is therefore desirable to cultivate mung bean germplasm with high yield and drought resistance to maintain the development of the mung bean industry. Publication of the mung bean genome [14] laid the foundation for molecular research in mung bean. Many studies have shown that NAC transcription factors have critical roles in plant growth and development, including in leaf and flower senescence [15,16,17], cell wall formation [18,19], fruit ripening [20,21,22], and root growth [23]. However, there have been few studies of NAC transcription factors in mung bean, and their functions in this economically valuable resource remain unexplored. Therefore, it is particularly important to identify important NAC transcription factors in mung bean, not only to enrich our understanding of these key transcription factors but also to lay a strong molecular foundation for the development of drought-resistant mung bean materials.

In this study, the transcriptome data obtained from mung bean were selected to analyze and predict the bioinformatics of the *VrNAC13* gene and the transcriptional initiation site and cis-acting element of the promoter of the *VrNAC13* gene. We then experimentally validated the transcriptional activation domain. Expression levels of *VrNAC13* in major mung bean tissues were measured with quantitative reverse transcription (qRT)-PCR. This study revealed novel information about the function of *VrNAC13* in the mung bean stress response and serves as a valuable reference for further study of NAC genes in mung bean.

## 2 Materials and methods

### 2.1 Test materials

Mung bean seeds of the cultivar “Yulv No.1” were provided by the Yulin University Agricultural Water Saving Research Group.

#### 2.1.1 Gene cloning materials

Healthy, plump seeds were sterilized with 5% sodium hypochlorite and then dried for later use. Individual pots were filled with 600 g of nutritious soil. Five seeds were sown in each pot. The soil moisture content was maintained between 70 and 80% by weight. For drought experiments, when the first trifoliate compound leaf was flattened, plants were divided into a control group and a treatment group. The control group had normal water management, whereas water was withheld from the treatment group. When the relative water content of the first trifoliate compound leaf in the treated sample significantly differed from that of the control group, the top leaf in the treatment group was collected and frozen in liquid nitrogen for further analysis.

#### 2.1.2 Gene expression analysis materials

(1) Tissue-specific gene expression analysis
  Plants were grown as described above. At the seedling stage, the roots, stems, and leaves were collected separately and frozen in liquid nitrogen for further analysis as described below.
(2) Gene expression analysis in drought-stressed seedlings
  Plants were grown as described above. The top 3 leaves of the first three leaves were collected from seedlings at four timepoints as described in Section 3.8.
(3) Gene expression analysis in ABA-treated seedlings
  Plants were grown as described above. At the seedling stage, 100 µmol/l ABA was sprayed on the top leaf of the first three leaves compound leaf. Leaves were collected at 0, 2, 4, 8, 12, and 24 h after treatment and flash-frozen in liquid nitrogen prior to further analysis. Samples collected at the 0 h timepoint served as the control.

### 2.2 *VrNAC13* cloning

Frozen mung bean leaves were ground to powder in liquid nitrogen [24]. RNA was extracted using a Transzol Kit (Gold, Beijing). RNA integrity was visualized with 1% agarose gel electrophoresis. cDNA was synthesized with a Transscript All In One First Strand cdnasvtheis Supermax for qPCR (One Step gDNA Removal) Reverse Transcription Kit [25]. oligo7 was used to design 21-bp primers specific for *VrNAC13* [26] (Table 1). The primers had no predicted secondary structure, low mismatch rates, and high specificity. Cloning was carried out via PCR using the mung bean cDNA as a template. The reactions were carried out in a 50-µl system containing 5 µl template cDNA (as required), 1 µl of each primers (10 μM), 5 µl buffer, 4 µl dNTPs (0.2 mM), 1 µl DNA polymerase(5units), and 33 µl nuclease-free water. The thermocycling protocol was as follows: pre-denaturation at 94°C for 3 min; 30 cycles of denaturation at 94°C for 15 s, annealing at 56°C for 15 s, and extension at 72°C for 1 min; and then 72°C for 7 min. Ultraviolet gel imager (Bio Rad) was used for imaging. Connect the PCR-amplified *VrNAC13* gene with the PMD-19T vector and then transform it into *Escherichia coli* DH5α. Conduct culture, then select monoclonal cells to propagate in liquid culture medium, use PCR to identify the strains, and after ensuring correct identification, and send them to Shanghai Biotechnology Co., Ltd. for sequencing. Extract plasmids from the correct sequencing bacterial solution and store them at −20°C.

**Table 1:** Primers used in this study

Primer name	Sequence (5–3′)	Purpose	Product length (bp)
VrNAC13-F	GAAGCTAGAACCGTGACCATC	Gene cloning	1,068
VrNAC13-R	CTAACCCAGTATCCACCCTAT		
Q-VrNAC13-F	GTTCCTCTTCCTGTCGCCATCATC	qRT-PCR	101
Q-VrNAC13-R	AAGTACCACTCTTGCTCGCCAAAC		
Vigna-actin-F	GTCGCACCACCAGAGAGGAAATAC	Internal reference gene	99
Vigna-actin-R	ATACTCAGCCTTCGCAATCCACATC		
ADfra1-F	CGGAATTCACGGGGGACTCTAGAATGAAT	Yeast transcriptional activation assay	458
ADfra1-R	CGGGATCCCTGCCAACAAGCCGGTACTCG		
ADfra12-F	CGGGATCCGAAGCTGAAACTGTTGCCTCA	Yeast transcriptional activation assay	623
ADfra2-R	CGGAATTCCGAGTACCGGCTTGTTGGCAG		
ADfra3-F	CGGAATTCACGGGGGACTCTAGAATGAAT	Yeast transcriptional activation assay	685
ADfra3-R	CGGGATCCTGGTGTTATTGTTGGCCATTG		
ADfra4-F	CGGAATTCACGGGGGACTCTAGAATGAAT	Yeast transcriptional activation assay	1,060
ADfra4-R	CGGGATCCGAAGCTGAAACTGTTGCCTCA		

### 2.3 Bioinformatics analysis

DNAMAN was used to analyze the nucleic acid composition and to translate the nucleic acid sequence to an aa sequence. The basic physical and chemical properties of *VrNAC13* were analyzed with DNAMAN software. The secondary protein structure was predicted with SOPMA (http://npsa-pbil.ibcp.fr/cgi-bin/npsa_automat.pl?page=npsa_sopma.html, accessed on 14 May 2022). The tertiary protein structure was predicted with Phyre2 (http://www.sbg.bio.ic.ac.uk/phyre2/html/page.cgi?id=index, accessed on 14 May 2022). Protein domain analysis was conducted on the NCBI website using the Conserved Domains Database (https://www.ncbi.nlm.nih.gov/Structure/cdd/wrpsb.cgi, accessed on 14 May 2022). Phosphorylation sites were predicted with NetPhos3.1 (http://www.cbs.dtu.dk/services/NetPhos/, accessed on 14 May 2022). All known NAC sequences in *Arabidopsis* were downloaded from The Arabidopsis Information Resource (https://www.arabidopsis.org/index.jsp, accessed on 14 May 2022). A phylogenetic tree was constructed in MEGA5.10 using the *Arabidopsis* sequences and *VrNAC13*. BDGP (https://www.fruitfly.org/seq_tools/promoter.html, accessed on 14 May 2022) was used to predict the transcription initiation site and putative core promoter regions in *VrNAC13*. *Cis*-acting elements in the promoter region of *VrNAC13* were predicted with PlantCARE (http://bioinformatics.psb.ugent.be/webtools/plantcare/html/, accessed on 14 may 2022) [27,28,29].

### 2.4 Verification of *VrNAC13* transcriptional activity

Based on the structural characteristics of *VrNAC13*, the gene was divided into four parts (fra1, fra2, fra3, and fra4). Primers were designed for each of these fragments (ADfra1, ADfra2, ADfra3, and ADfra4, respectively) (Table 1). Each of the corresponding target fragments was amplified using these primers, and then they were inserted into the pGBKT7-BD vector (linearized with EcoRI and BamHI). The resulting recombinant vectors were named pGBKT7-fra1, pGBKT7-fra2, pGBKT7-fra3, and pGBKT7-fra4, respectively. *E. coli* was transformed with each vector separately to generate four strains, each containing a plasmid with one fragment. Transformants were screened with kanamycin, positive colonies were selected, and the target plasmid was extracted. The target plasmids were each transferred into competent yeast cells (Clontech) following the manufacturer’s instructions. The strains containing the pGBKT7-p53 + pGADT7-largeT and the pGBKT7-LamC + pGADT7-largeT vectors were used as the positive and negative controls, respectively. Transformed yeast cells were grown on solid SD/-Trp/+ 5Mm3-AT medium for three days. After colonies had grown, X-Gal was added and interactions were determined based on colony color.

### 2.5 *VrNAC13* expression analysis

To determine tissue-specific *VrNAC13* expression, the top leaves of the first three compound leaves and the roots and stems were collected from untreated plants. For abiotic stress experiments, samples were collected as described above. For all samples, RNA was extracted and cDNA was generated as described above. qRT-PCR was conducted on a CFX96 Real-Time System (Bio-Rad) using *VrNAC13*-specific primers (Table 1). The mung bean *ACTIN* gene served as the internal reference for gene expression normalization. *VrNAC13* expression levels were normalized using the 2^−ΔΔCt^ method [30].

## 3 Results

### 3.1 Cloning and sequence analysis of *VrNAC13*

**Figure 1:**
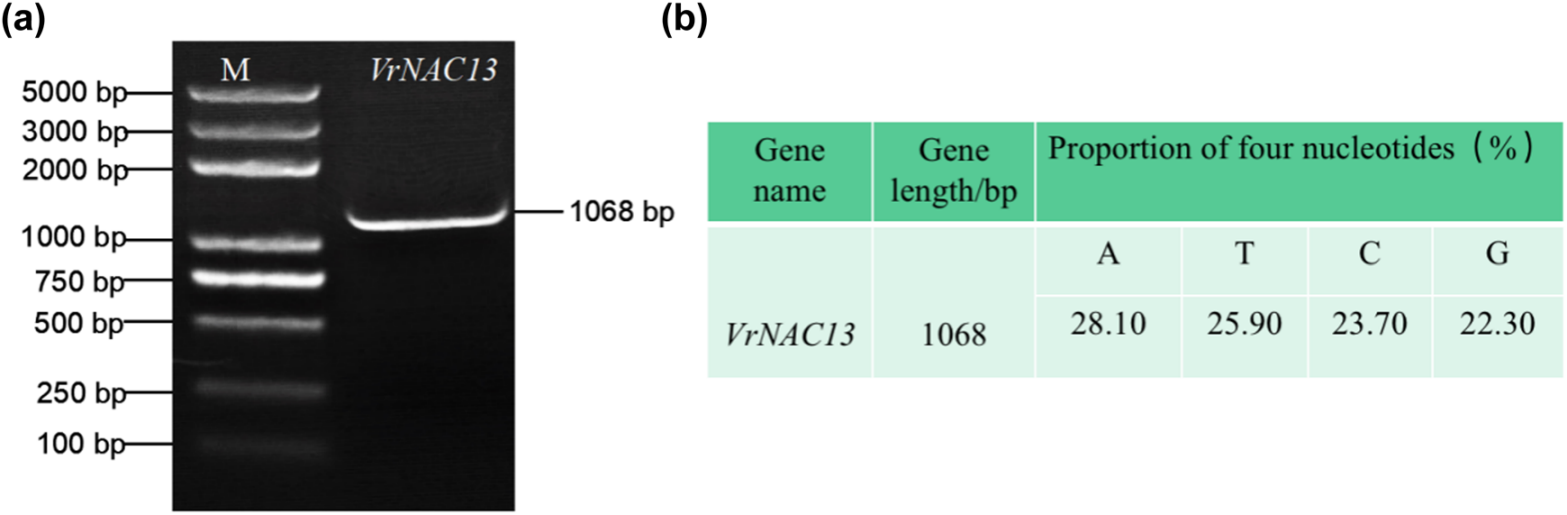
Analysis of *VrNAC13* structure and content. (a) Visualization of amplified *VrNAC13* with 1% agarose gel electrophoresis. M, Trans2K Plus DNA Marker. (b) Nucleotide content of *VrNAC13* as determined by sequencing.

*VrNAC13* was sequenced and determined to be 1,068 bp in length (Figure 1a). The start codon was ATG and the stop codon was TAG. The nucleotide content was 28.1% A, 25.9% T, 23.7% C, and 22.3% G. There were no unresolved nucleotides in the sequence (Figure 1b).

### 3.2 Physical and chemical properties of *VrNAC13*

**Figure 2:**
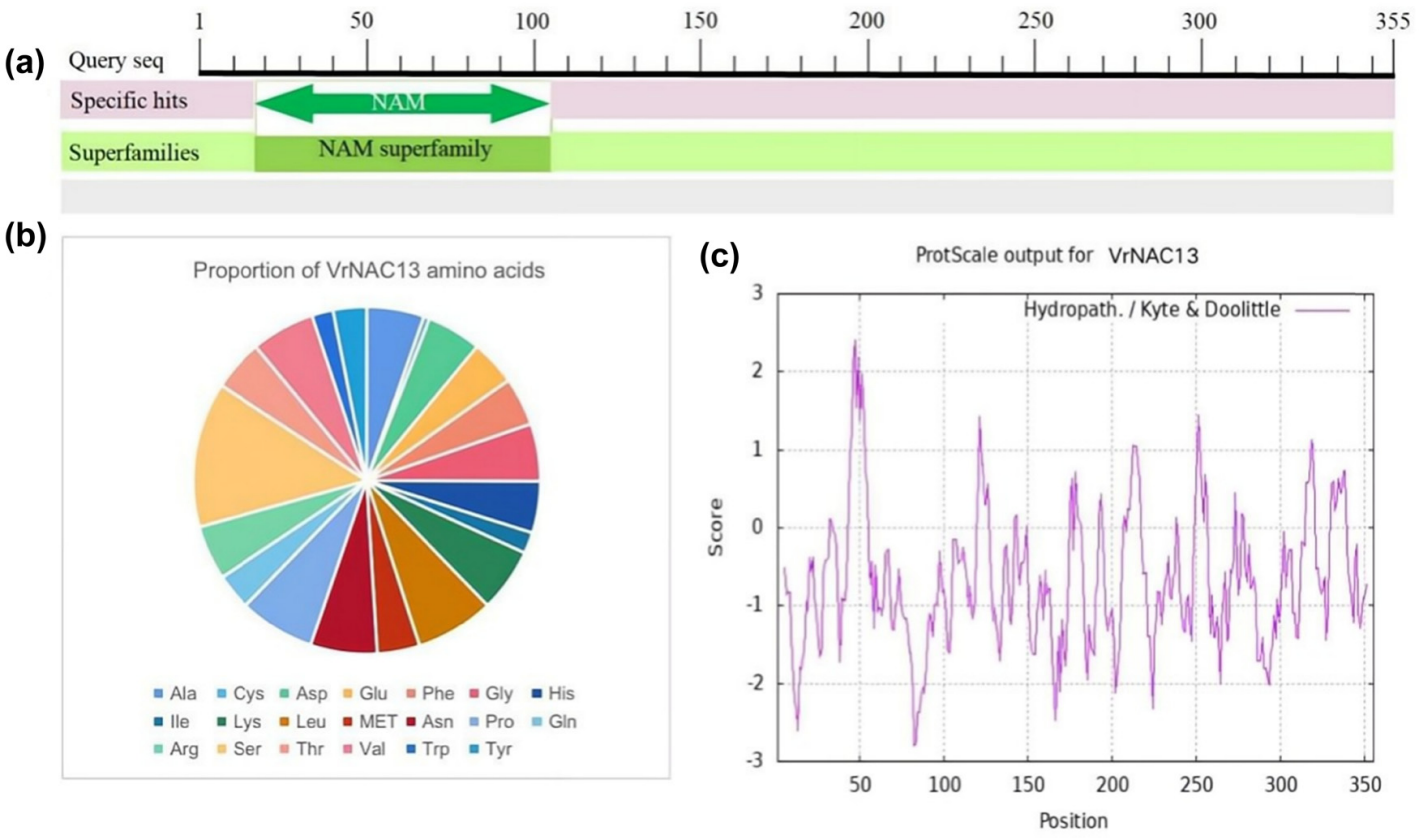
Physical and chemical properties of *VrNAC13*. Predictions of the conserved domains (a), amino acid composition (b), and hydrophobicity of each region (c) in *VrNAC13*.

The amino acid sequence of *VrNAC13* was next analyzed (Figure 2). A NAM domain was predicted, comprising residues 19–145 (Figure 2a). *VrNAC13* was 355 amino acids (aa) in length (Figure 2b) with a molecular weight of 39.9 KDa and an isoelectric point of 9.51. Ser was the most abundant residue at 13.52%, whereas Cys was the least abundant (0.56%). The protein contained 39 positively or negatively charged residues (Arg or Lys and Asp or Glu, respectively). ProtScale was used to analyze the hydrophilic and hydrophobic properties of *VrNAC13* (Figure 2c). The lower the score, the stronger the hydrophilicity; the higher the score, the stronger the hydrophobicity. The protein encoded by *VrNAC13* is in the region with score <0, which is significantly more than that with score >0. It is predicted that *VrNAC13* is a hydrophilic protein.

### 3.3 Prediction and analysis of phosphorylation sites in *VrNAC13*

**Figure 3:**
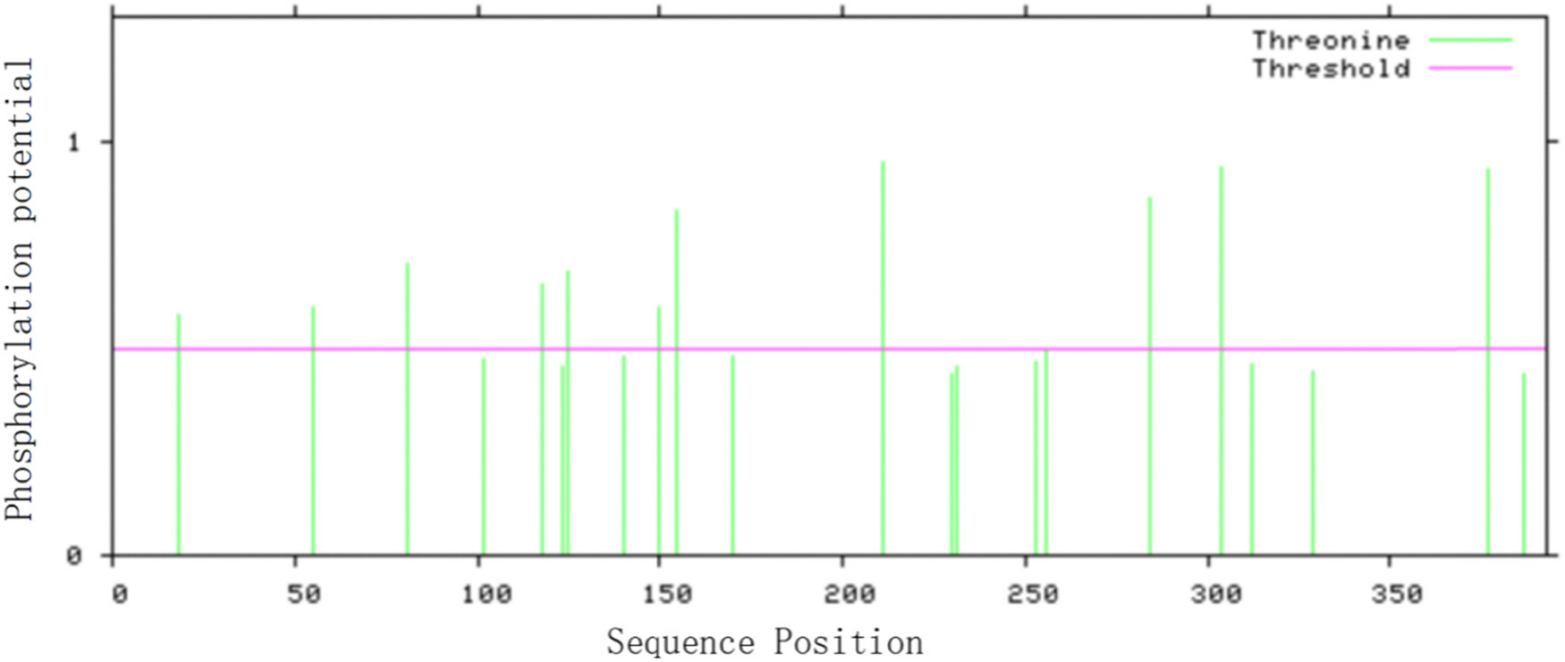
Predicted phosphorylation sites within *VrNAC13*.

Phosphorylation sites in *VrNAC13* were predicted with NetPhos3.1. The protein was predicted to contain 11 threonine phosphorylation sites (Figure 3).

### 3.4 Predicted secondary and tertiary structures of *VrNAC13*

**Figure 4:**
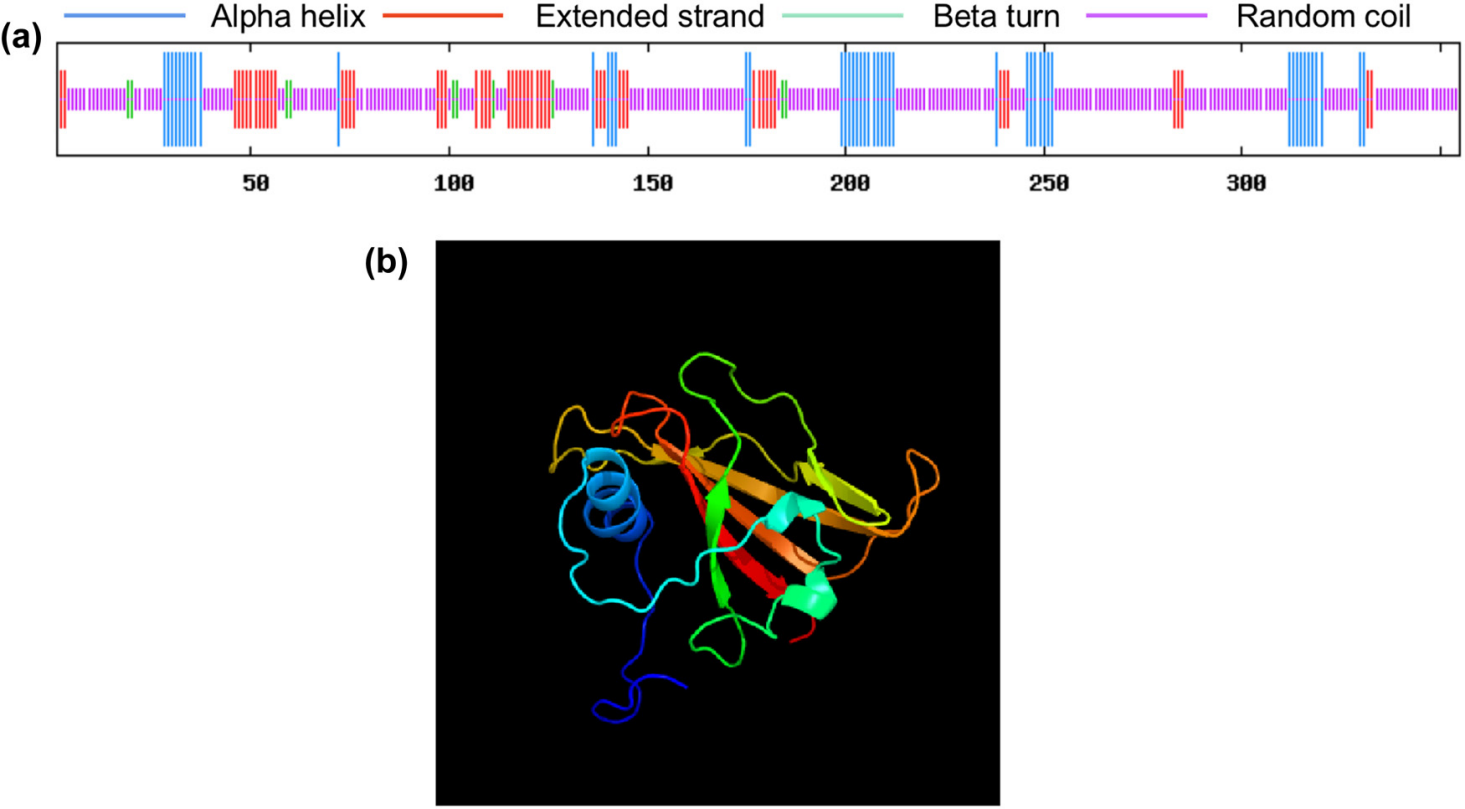
Predicted secondary (a) and tertiary (b) structures of *VrNAC13*.

SOPMA and Phyre2 were used to predict the structure of *VrNAC13* at the secondary and tertiary levels (Figure 4). The secondary structure of *VrNAC13* was predicted to be 14.08% α-helixes (50 aa), 67.04% random coils (238 aa), 16.06% extended chains (57 aa), and 2.82% β-turns (10 aa) (Figure 4a). The main spatial structure of *VrNAC13* protein is composed of α-Helix and irregular curl constitute, which is consistent with the secondary structure prediction analysis.

### 3.5 Phylogenetic analysis of *VrNAC1*3

**Figure 5:**
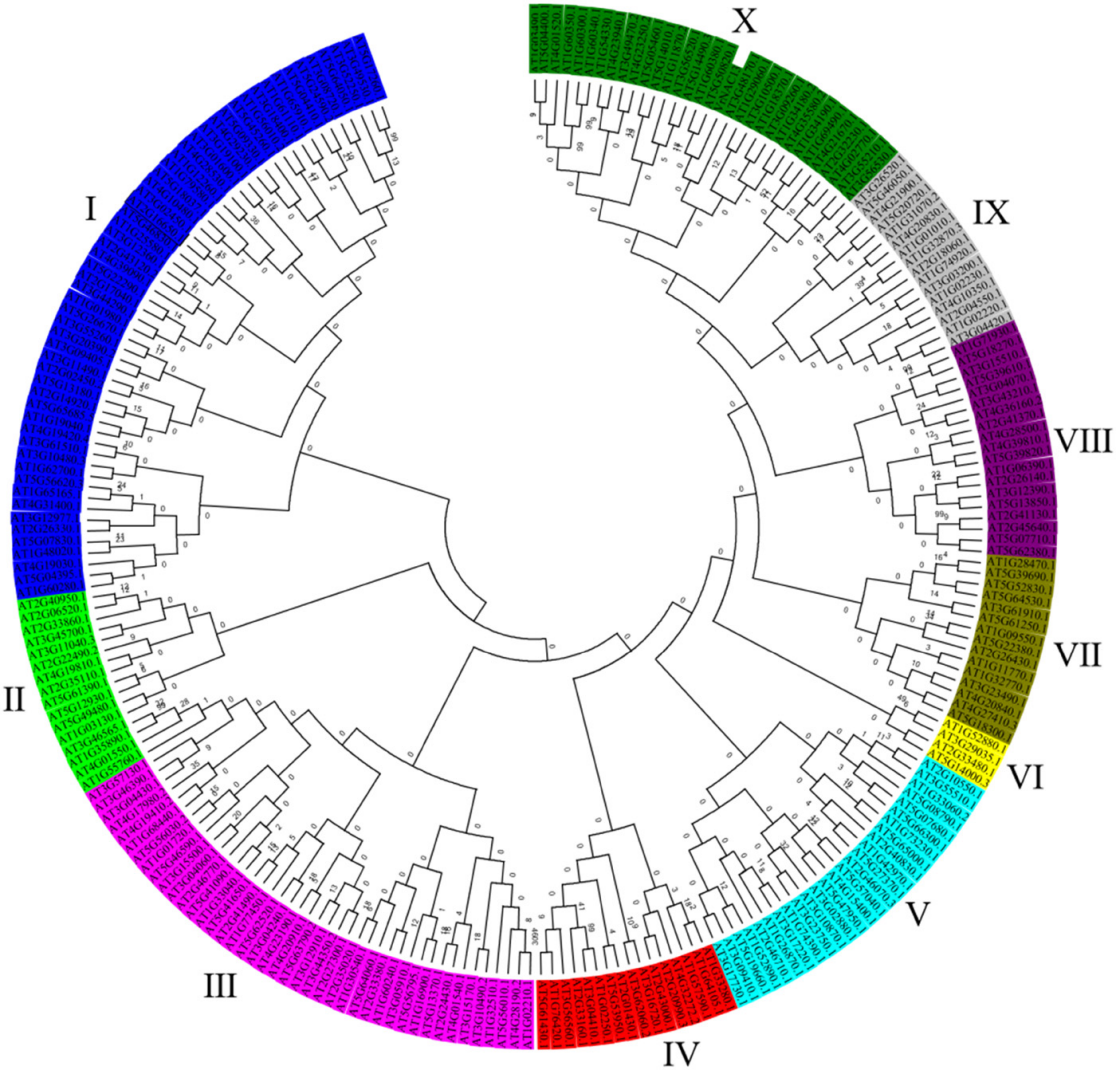
Phylogenetic analysis of *VrNAC13* and *Arabidopsis thaliana* NAC proteins.

Amino acid sequences of all predicted NAC proteins in *Arabidopsis* were collected, totaling 241 proteins. These sequences and *VrNAC13* were used to construct a phylogenetic tree in MEGA5.10 using the maximum likelihood method. The 242 proteins were grouped into 10 categories (labeled I–X) (Figure 5). *VrNAC13* and 31 *Arabidopsis* NAC proteins clustered together in subclass *Ⅹ*. The genes in each subclass had similar predicted functions, and we therefore hypothesized that *VrNAC13* was similar in function to the proteins encoded by AT1G60380.1 and AT5G50820.1, which had the highest similarity scores with *VrNAC13*.

### 3.6 Verification of transcriptional activation in *VrNAC13*

**Figure 6:**
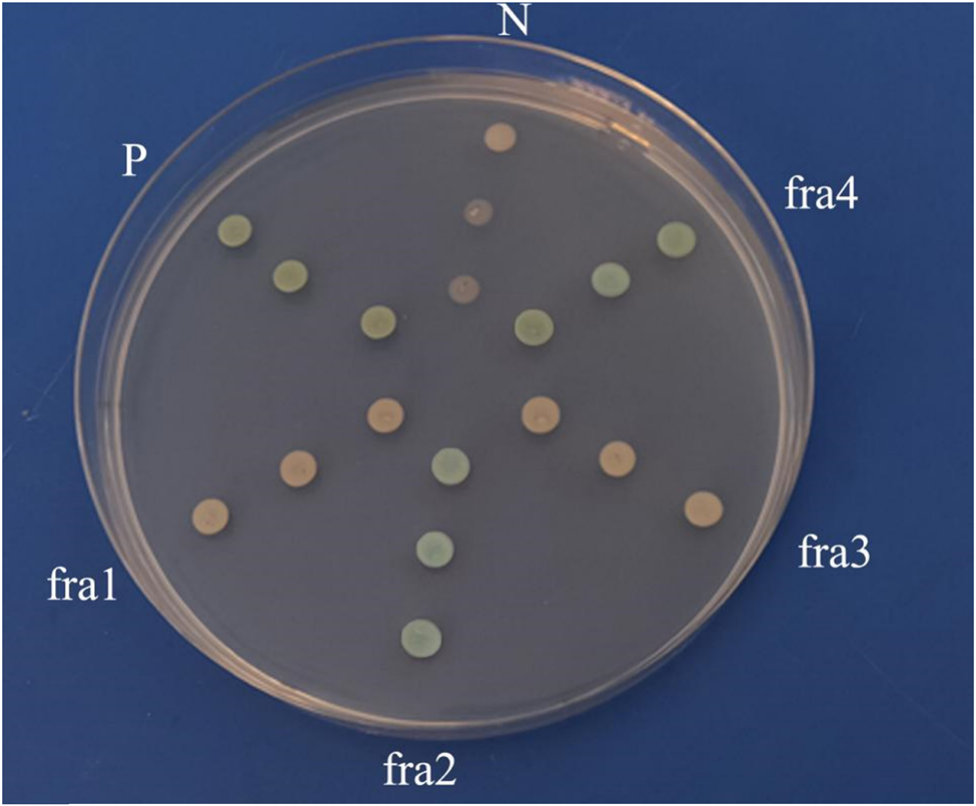
Analysis of the transcriptional activation activity of *VrNAC13*. Yeast was transformed with the recombinant vectors pGBKT7-fra1, pGBKT7-fra2, pGBKT7-fra3, and pGBKT7-fra4. pGBKT7-53 + pGADT7-largeT (P) was the positive control and pGBKT7 -LamC + pGADT7-largeT (N) was the negative control.

Conserved domain analysis indicated that *VrNAC13* was divided into four parts: the first, from nucleotides 1 to 458, encoded the NAM domain. The second spanned positions 437–1,060 and was the sequence from which the NAM domain was removed. The third component was from positions 1 to 685 and contained C-terminal sequences on the basis of the NAM domain. The fourth part, from position 1 to position 1060, comprised the entire ORF. The positive control was blue when grown on SD/-Trp/X-α-gal + 5 mM 3-AT medium. Yeast transformed with the pGBKT7-fra2 or pGBKT7-fra4 vectors was also blue, indicating that *VrNAC13* had an active domain with transcriptional activation activity (Figure 6).

### 3.7 Sequence analysis of the *VrNAC13* promoter

BDGP was used to predict the transcription initiation site of *VrNAC13*. This analysis revealed three potential core promoter regions, located from −955 to −905 bp, from −875 to −825 bp, and from −137 to −87 bp. The associated scores were 0.89, 0.87, and 0.92, respectively, and the possible transcription initiation sites were A, G, and A, respectively, the higher the score, the greater the possibility that the region is the core promoter region. The region from −137 to −87 bp had the highest score in addition to a TATA box ∼20–30 bp upstream and a CAAT box ∼70–80 bp upstream, indicating that that region was the most likely core promoter region of the gene; the transcription initiation site was at 2,362 bp. The *cis*-acting element prediction tool PlantCARE was used to analyze *VrNAC13*, and this analysis showed that the promoter contained not only core elements (such as the CAAT-box and the TATA-box) but also response elements for hormones such as abscisic acid (ABRE element) and gibberellin (GAREmotif). Predicted *cis*-acting elements, including stress response elements (LTRs) and light response elements (AREs), are shown in Table 2.

**Table 2:** Analysis of predicted *cis*-acting elements in the *VrNAC13* promoter

Element type	Element name	Copy number	Motif sequence	Function
Basal element	TATA-box	2	TATA/ATATAA/TATACA	Core promoter element
	CAAT-box	16	CAATT/CAAT/CCAAT	Common *cis*-acting element in promoter and enhancer regions
Phytohormone response	ABRE	1	ACGTG	*Cis*-acting elements involved in abscisic acid reaction
	CGTCA-motif	1	CGTCA	*Cis*-acting regulatory element involved in methyl jasmonate (MeJA) responsiveness
	TGACG-motif	1	TGACG	*Cis*-acting regulatory element involved in the MeJA-responsiveness
Light response	G-box	1	TACGTG	*Cis*-acting regulatory element involved in light responsiveness
	AE-box	1	AGAAACTT	Component of light response module
	GATA-motif	1	GATAGGA	Component of light-responsive element
	GT1-motif	1	GGTTAA	Light-responsive element
	CAG-motif	1	GAAAGGCAGAC	Component of light-response element
Stress response	LTR	1	CCGAAA	*Cis*-acting element involved in low-temperature responsiveness
	ARE	1	AAACCA	*Cis*-acting regulatory element essential for anaerobic induction
Other	ABRE3a	1	TACGTG	
	WRE3	2	CCACCT	
	ABRE4	1	CACGTA	
	W box	1	TTGACC	
	as-1	1	TGACG	
	box S	1	AGCCACC	

### 3.8 Response of *VrNAC13* to drought and ABA stress

**Figure 7:**
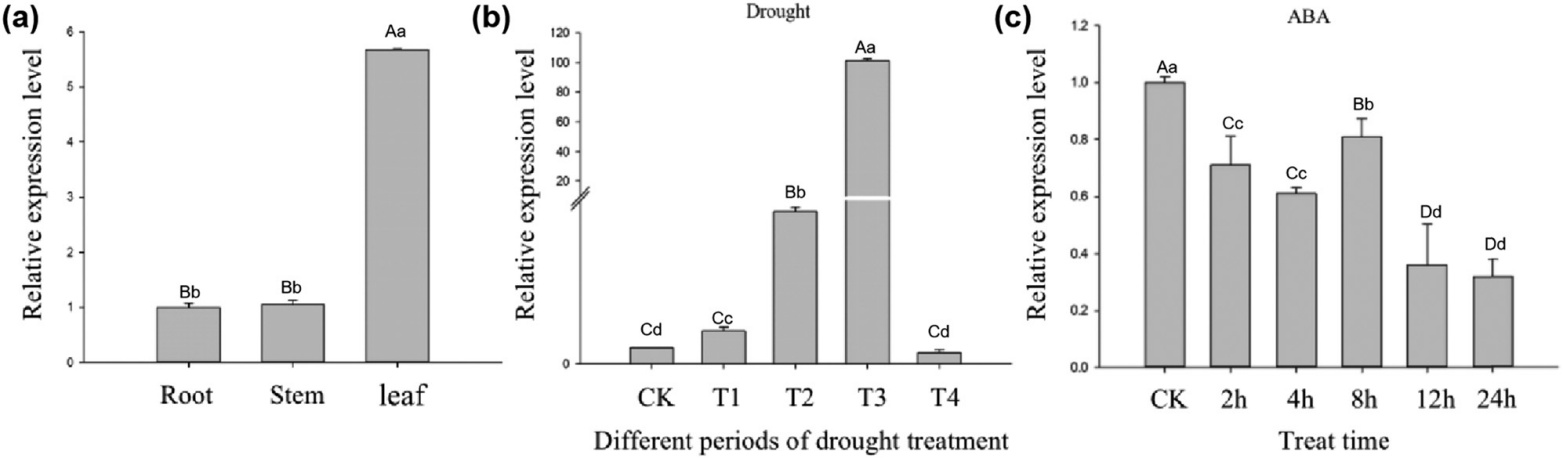
Expression levels of *VrNAC13* in multiple tissues and after treatment with abiotic stressors. (a) *VrNAC13* expression in three mung bean tissues. (b) *VrNAC13* expression in response to drought. CK, control (untreated). T1, the stage in which there were clear differences in stomatal conductance between the leaves of the drought-stressed group and the control group. T2, the stage in which there were clear differences in relative leaf water content between the drought-stressed group and the control. T3, the stage in which mung bean leaves were visibly wilted. T4, the stage after rehydration treatment. (c) *VrNAC13* expression in response to exogenous treatment with 100 µmol/l abscisic acid (ABA). Lowercase and uppercase letters indicate statistical significance groups at *p* < 0.05 and *p* < 0.01, respectively.

To clarify the function of *VrNAC13*, expression levels of this gene in three different tissues and in response to stress conditions were analyzed with qRT-PCR (Figure 7). *VrNAC13* was found to be specifically expressed in the leaves, with relatively low expression in the roots and stems. Drought stress was carried out and analyzed at several stages: T1, the stage in which there were clear differences in stomatal conductance between the leaves of the drought-stressed group and the control group; T2, the stage in which there were clear differences in relative leaf water content between the drought-stressed group and the control; T3, the stage in which mung bean leaves were visibly wilted; and T4, the stage in which plants were re-hydrated. Drought stress was found to significantly promote *VrNAC13* expression in the leaves, with relative expression levels peaking at T3. There were no significant differences in *VrNAC13* levels between the control and T4 plants. Expression levels were also assessed at several timepoints after treatment with ABA, an important stress hormone that regulates plant physiological processes and drought responses. There were significant differences in *VrNAC13* expression between timepoints after ABA treatment; *VrNAC13* levels first decreased and then increased again, peaking at 8 h at a level below that of the untreated control. This indicated that *VrNAC13* was induced by drought and inhibited by ABA treatment (Figure 7).

## 4 Discussion

Non-biotic stress triggers a wide range of plant responses, including gene expression, cell metabolism, plant growth and development, and crop yield. In recent years, multiple transcription factors have been confirmed to participate in the regulation of growth and development, defense regulation, and stress response in different types of plants [31,32,33,34]. As one of the largest TF families in plants, NAC has been proven to play an important role in drought stress [35,36,37,38].

Members of the NAC transcription factor family have important roles in numerous plant processes. Studies have shown that these genes play key roles in plant growth, development, stress responses, and specialized metabolite biosynthesis. It is of great significance to study the roles of NAC genes in plant growth and development and in responses to biotic and abiotic stresses, ultimately to develop plants that can more effectively resist drought. In the present study, *VrNAC13* was cloned from mung bean. Sequence analysis showed that *VrNAC13* was 1,068 bp in length and contained 28.1% A, 25.9% T, 23.7% C, and 22.3% G content. There were no unresolved nucleotides from the sequencing process. The gene encodes 355 amino acids, and conserved domain analysis revealed the presence of a conserved NAM domain and an AD transcriptional activation domain at the C-terminal (from nucleotide positions 437–1,060). The latter was validated with a yeast transcriptional activation experiment. This is consistent with previous research on NAC transcription factors such as Chickpea (*Cicer arietinum* L.) [39] and Soybean (*Glycine max*) [40] and conforms to the structural characteristics of NAC transcription factors. *VrNAC13* was predicted to have 11 threonine phosphorylation sites, suggesting that this protein may be modified and regulated through phosphorylation. Phylogenetic analysis of *VrNAC13* and *Arabidopsis* NAC *VrNAC13* shared the highest levels of similarity with AT1G60380.1 and AT5G50820.1, and these two *Arabidopsis* proteins are primarily involved in transcriptional regulation, indicating that *VrNAC13* may have a similar function in mung bean.

The promoter region is an important component in gene expression regulation. *Cis*-acting elements are specific sequences in the promoter that are bound by transcription factors. The type, number, sequence, and distance to other *cis*-elements affect their efficiency and strength in modulating the gene expression [41]. Analysis of the *VrNAC13* promoter region revealed the presence of numerous *cis*-acting elements related to abiotic stress and responses to hormones, including ABA and methyl jasmonate (MeJA). Changes in ABA levels activate many stress-response genes that function to induce stomatal closure, thus reducing transpiration and maintaining internal water levels [42]. Numerous studies have shown that there are certain differences in the expression patterns of NAC transcription factor genes in different plants. For example, soybean *GmNAC1* is mainly expressed in roots and flower buds, while *GmNAC2* is strongly expressed in leaves, stems, and seeds, and weakly expressed in roots, flower buds, and pods. *GmNAC3* is highly expressed in leaves, flower buds, and pods, but relatively low in seed development [43]. *VrNAC13* is highly expressed in leaves after drought treatment, and we speculate that this gene may be involved in material transport during plant growth. *VrNAC13* showed significant expression changes over time after ABA treatment. Overall, compared to the control sample, *VrNAC13* was inhibited in the ABA-treated sample, which is consistent with previous research on tartary buckwheat *FtNAC11* [44]. These expression dynamics and the presence of an ABA-responsive *cis*-element in the *VrNAC13* promoter suggested that *VrNAC13* regulated the responses of mung bean leaves to drought via the ABA pathway.

At present, northern China is facing increasingly common drought conditions. As a result, there is heightened dependence on molecular biological methods of crop management. It is particularly important to identify and clone drought-resistance genes to allow their transfer into drought-sensitive crops. This will allow researchers to cultivate new drought-resistant transgenic crop varieties that can not only make use of arid soil but also contribute to water-saving efforts and sustainable agricultural development. We here analyzed the basic structure of *VrNAC13* at the nucleic acid and protein levels and predicted its function as a drought-response gene. Future experiments should further explore the function of *VrNAC13* through exogenous expression in another system (e.g., *Arabidopsis*) and subsequent observation of transgenic plant responses to a range of stressors. This would allow preliminary verification of *VrNAC13* function and ultimately clarify the stress resistance mechanism in which *VrNAC13* functions, laying a molecular foundation for stress-resistant mung bean breeding.

## 5 Conclusion

This study cloned *VrNAC13* and analyzed its structure. Based on qRT-PCR, the tissue expression specificity and expression after different treatments were analyzed to elucidate the structural and functional characteristics of the *VrNAC13* gene, laying a foundation for drought resistance molecular breeding in mung beans.
